# Baseline clinical and MRI risk factors for hamstring reinjury showing the value of performing baseline MRI and delaying return to play: a multicentre, prospective cohort of 330 acute hamstring injuries

**DOI:** 10.1136/bjsports-2023-107878

**Published:** 2024-05-10

**Authors:** Muhammad Ikhwan Zein, Milo J K Mokkenstorm, Marco Cardinale, Louis Holtzhausen, Rod Whiteley, Maarten H Moen, Guus Reurink, Johannes L Tol

**Affiliations:** 1 Department of Orthopedic Surgery and Sports Medicine, Amsterdam UMC Location AMC, Amsterdam, The Netherlands; 2 Faculty of Sports Science, Universitas Negeri Yogyakarta, Yogyakarta, Special Region of Yogyakarta, Indonesia; 3 Sports, Amsterdam Movement Sciences, Amsterdam, The Netherlands; 4 Sports Science, Aspetar Orthopaedic and Sports Medicine Hospital, Doha, Ad Dawhah, Qatar; 5 Sports Medicine Department, Aspetar Qatar Orthopaedic and Sports Medicine Hospital, Doha, Qatar; 6 Section Sports Medicine, Faculty of Health Sciences, University of Pretoria, Pretoria, South Africa; 7 Aspetar Orthopaedic and Sports Medicine Hospital, Doha, Ad Dawhah, Qatar; 8 Department of Sports Medicine, Bergman Clinics BV, Naarden, The Netherlands; 9 Amsterdam Movement Sciences (AMS), Academic Center for Evidence Based Sports Medicine (ACES), Amsterdam UMC Location AMC, Amsterdam, The Netherlands

**Keywords:** Hamstring Muscles, Risk factor, Sports medicine, Sporting injuries, Recurrence

## Abstract

**Objectives:**

Studies identifying clinical and MRI reinjury risk factors are limited by relatively small sample sizes. This study aimed to examine the association between baseline clinical and MRI findings with the incidence of hamstring reinjuries using a large multicentre dataset.

**Methods:**

We merged data from four prospective studies (three randomised controlled trials and one ongoing prospective case series) from Qatar and the Netherlands. Inclusion criteria included patients with MRI-confirmed acute hamstring injuries (<7 days). We performed multivariable modified Poisson regression analysis to assess the association of baseline clinical and MRI data with hamstring reinjury incidence within 2 months and 12 months of follow-up.

**Results:**

330 and 308 patients were included in 2 months (31 (9%) reinjuries) and 12 months (52 (17%) reinjuries) analyses, respectively. In the 2-month analysis, the presence of discomfort during the active knee extension test was associated with reinjury risk (adjusted risk ratio (ARR) 3.38; 95% CI 1.19 to 9.64). In the 12 months analysis, the time to return to play (RTP) (ARR 0.99; 95% CI 0.97 to 1.00), straight leg raise angle on the injured leg (ARR 0.98; 95% CI 0.96 to 1.00), the presence of discomfort during active knee extension test (ARR 2.52; 95% CI 1.10 to 5.78), the extent of oedema anteroposterior on MRI (ARR 0.74; 95% CI 0.57 to 0.96) and myotendinous junction (MTJ) involvement on MRI (ARR 3.10; 95% CI 1.39 to 6.93) were independently associated with hamstring reinjury.

**Conclusions:**

Two clinical findings (the presence of discomfort during active knee extension test, lower straight leg raise angle on the injured leg), two MRI findings (less anteroposterior oedema, MTJ involvement) and shorter time to RTP were independently associated with increased hamstring reinjury risk. These findings may assist the clinician to identify patients at increased reinjury risk following acute hamstring injury.

**Trial registration numbers:**

NCT01812564; NCT02104258; NL2643; NL55671.018.16

**WHAT IS ALREADY KNOWN ON THIS TOPIC**

- Several clinical findings are associated with hamstring reinjury risk.
- The evidence for MRI findings and their association with hamstring reinjury risk is limited.

**WHAT THIS STUDY ADDS**

- Baseline clinical and MRI findings are valuable for identifying hamstring reinjury risk factors.
- The baseline clinical findings (presence of discomfort during active knee extension test and lower straight leg raise angle on the injured leg), MRI findings (less extent of oedema anteroposterior and myotendinous junction involvement) and time to return to play are newly identified reinjury risk factors.
- The presence of discomfort during the active knee extension test is associated with hamstring reinjury risk within both 2-month and 12-month follow-up.
- Previous hamstring injury that has previously been identified as a risk factor for reinjury was not a strong predictor in this study.

**HOW THIS STUDY MIGHT AFFECT RESEARCH, PRACTICE OR POLICY**

- As part of the routine clinical examination, the presence of discomfort during the active knee extension test and a lower straight leg raise angle on the injured leg should be considered in the return to play decision-making and risk management process after acute hamstring injury.
- MRI at initial injury provides valuable information on profiling reinjury risk in athletes.
- Delaying time to return to play might reduce the reinjury risk.

## Introduction

Hamstring injuries are the most common injury in many sports and have a high reinjury rate in both professional and recreational athletes (14%–63%).^1–7^ Despite increased attention to researching treatment, prevention and suggested protocols for injury reduction,^8^ the incidence and time loss caused by hamstring injuries has increased over the last 20 years, with one out of five hamstring injuries being a reinjury.^4^ Hamstring reinjuries are associated with a longer time to recovery than the initial injury^7^ and lead to increased risk for further reinjury.

Secondary prevention strategies rely on the identification of risk factors for hamstring reinjuries in order to mitigate and reduce their occurrence. A recent meta-analysis reported that several clinical findings, such as older age, previous hamstring injuries, a history of anterior cruciate ligament injury and a history of calf strain, were associated with an increased reinjury risk.^9^ For imaging findings, three previous systematic reviews have found limited to moderate evidence for four baseline MRI findings to represent risk factors for reinjury^2 10 11^: grade 1 hamstring injury, a larger volume of the initial injury, located at biceps femoris and intratendinous injuries. However, there were several risks of bias from the studies included in these analyses, such as a lack of consistency in reinjury definition, heterogeneous risk factors and study methods, unrepresentative subjects and no adjustment for confounding factors. A study on MRI findings shortly prior to return to play (RTP) by Isern-Kebschull *et al* showed that the presence of two of these five radiological signs was associated with increased reinjury risk; connective tissue gap, loss of tendon tension, intermuscular oedema, callus gap and interstitial feather oedema.^12^

A main limitation of the existing studies in the literature is that sample sizes of reinjuries are too small to detect possibly clinically relevant associations between clinical and imaging factors and reinjury risk.^9 11^ About 30–50 reinjury cases are needed to detect a moderate to strong association between risk factors and reinjury risk,^13^ and a multivariable analysis approach would require an even larger sample size. A previous prospective study from our group with 17 reinjury cases did not allow an adequately powered multivariable analysis.^1^ Considering the limitations of small sample sizes to understand the aetiology of hamstring muscle reinjuries, we have combined four prospective cohorts of patients with an acute hamstring injury registered in different studies at different centres.

The aim of this study was to examine the association between baseline clinical and MRI findings with the incidence of hamstring reinjury within 2 months and 12 months in a much larger sample and more predictor findings. We hypothesised that such an approach could identify commonly performed clinical and MRI findings associated with increased reinjury risk, which have not been identified in previous studies with smaller sample sizes, and provide improved insights on hamstrings reinjuries.

## Methods

### Patients

In these analyses, we combined data from four prospective studies conducted in Qatar and the Netherlands: three randomised controlled trials regarding the effect of injectable agents following hamstring injury (Growth Factor study, ClinicalTrials.gov NCT 01812564^14^; Hamstring Injection Therapy Study, Dutch Trial Register NL2643^15^ and Rehabilitation of Acute Hamstring Injury study, ClinicalTrials.gov NCT 02104258^16^) and one ongoing prospective case series study aiming to evaluate the ability of MRI diffusion tensor imaging (DTI) to detect hamstring muscle injury and its correlation with the convalescent period and RTP (DTI for Hamstring Injury study, CCMO NL55671.018.16).

All studies included participants with a clinical diagnosis of recent hamstring injury in combination with a grade 1 or 2 (modified Peetrons grading system) hamstring lesion on MRI. All patients in the completed studies underwent a standardised rehabilitation protocol that has been described in detail in previous publications under the supervision of experienced sports physiotherapists.^14–16^ The patients of the ongoing study (DTI for hamstring injury) were advised to be treated using a criteria-based rehabilitation programme but on a voluntary basis. There were differences in the standardised rehabilitation protocol performed in the four studies. An overview of the study design of the included studies (including eligibility criteria, study intervention and rehabilitation protocols) can be found in online supplemental appendices 1 and 2.

10.1136/bjsports-2023-107878.supp1 Supplementary data

The clinical trials took place from February 2014 to February 2023.

### Equity, diversity and inclusion statement

The population of this study was athletes of all genders, races/ethnicities and all levels of play (professional and non-professional) with acute hamstring injury in Qatar and the Netherlands. Thus, findings may not be generalisable to settings with fewer resources. Our study group consisted of women and men from different nationalities (European and Asian countries) with different disciplines (sports physician, orthopaedic, physiotherapist, human movement sciences and statistician), including junior scholars.

### Baseline data collection and selection of variables for analysis

All baseline assessment variables were collected on the same day of inclusion before administration of any injection or treatment, except the variable ‘time to RTP’, which recorded the number of days from the initial injury until the patient was cleared to resume unrestricted training (RTP). Variables were selected for analysis if they were included in all four of the original studies included.

For the current analyses, we obtained baseline information about age, gender (male or female), height (centimetres), weight (kg), body mass index (kg/m^2^), date of injury, time since injury (days), type of sports, level of sports (professional or non-professional), type of injury (sprinting or non-sprinting), side of hamstring injury (left or right) and history of hamstring injury (yes or no).

The clinical examination included hamstring flexibility testing, isometric strength testing, and muscle palpation. The flexibility test was assessed with the passive straight leg raise test and active knee extension test.^1 17 18^ For the passive straight leg raise test, the participant positioned supine, and the researcher raised the participant’s leg with an extended knee until maximal tolerable stretch while the contralateral leg remained flat on the table. At the endpoint of maximal tolerable stretch, the angle between the leg and the table (in degree) was measured. Active knee extension test was performed with the participant positioned supine, 90^o^ hip flexion of the tested leg. The participant was instructed to extend the tested knee until the maximum tolerable stretch, with the contralateral leg remaining flat on the table. At the endpoint of maximal tolerable stretch, the absolute knee angle (in degrees) was measured. Participants were also asked to report if they experienced localised pain during the test. Both passive straight leg test and active knee extension test were performed once by the researcher.

The isometric strength test was measured using a handheld dynamometer (Hoggan MicroFET2; Hoggan Scientific, Salt Lake City, Utah, USA) in 15° and 90° of knee flexion,^1 19^ and recorded in Newtons (N). The palpation technique was performed to measure the length of painful area (centimetres) as described by Askling *et al*.^20^

MRI was performed using comparable protocols, including sequences that are suitable for detecting muscle injury. Three RCTs used a 1.5-Tesla (T) MRI, and the current ongoing cohort study has been collecting and analysing images with a 3.0 T MRI. The MRI protocols of the studies have been described in detail in previous publications.^14 16 21 22^ MRIs were scored by one out of four experienced musculoskeletal radiologists (EA, FFS, SB and MM) using a standardised data collection form.^23 24^ Good to excellent intraobserver and interobserver reliability for MRI parameters measures were previously described.^25^ The following identical MRI parameters were identified across all four studies: muscle involved (biceps femoris or semimembranosus/semitendinosus), tendon involvement (no tendon involved or tendon involved), the myotendinous junction (MTJ) involved (no MTJ involved or MTJ involved), the extent of oedema (centimetres), the extent of haematoma (centimetres), grade of injury (grade 1 or 2), intramuscular (IM) tendon disruption (no IM disruption or IM disruption), total IM tendon disruption (no disruption/partial disruption or total disruption), waviness (present or absent) and fibrosis (present or absent).

### Data inspection and assessment

#### Data merging

Data merging was performed to combine the data recorded into a single dataset. The dataset across four studies was accessed from an anonymised online database system, which met the safety criteria and standards of good clinical practice. The new source dataset (master data file) was created to pool all variables from each study. During the process, two researchers (MJKM and MIZ) checked for any differences in values from the data sources to the merged data to ensure veracity. Any differences in categories or values measured between studies were discussed in the research group to reach a consensus for recoding. A final check was performed to ensure that data values in the master data file were complete and identical to the source data record from each study. All data were anonymised at the source before being included in the database.

#### Data cleaning

To detect and reduce the chance of any error during the process of data merging, a data cleaning protocol was independently conducted by two researchers (MJKM and MIZ). The data cleaning protocol was constructed based on the guidelines of the Department of General Practice of the Erasmus MC (Rotterdam, The Netherlands). The protocol included manually checking all data for odd data points or chronological inconsistencies and all derived variables for correctness. Additionally, all measurements of a random selection of 3% of all participants (per original database) were manually checked for consistency with the original measurements. If the percentage of error exceeded 1.5%, the random selection of participants was increased to 15% in case the threshold of error (1.5%) was exceeded, all data had to be digitally rescanned and reprocessed. A detailed description of the data cleaning protocol can be found in online supplemental appendix 3.

### Primary outcome

The primary outcome measures were the occurrence of a reinjury within 2 months and 12 months after RTP. The definition and incidence of reinjury was based on the original studies. Reinjury was defined as the acute onset of posterior thigh pain in the same site/side. In the Hamstring Injection Therapy study, the injury had to result in absence from play to be classified as reinjury.^26^ All patients were contacted periodically by the investigators of the original studies. They were also instructed to contact the principal investigator in any case of suspected reinjury.

### Statistical analysis

Statistical analyses were conducted by using SPSS software (V.28.0; SPSS). We analysed baseline patient characteristics using descriptive analysis. The descriptive data were presented as mean (SD) or median (IQR) for continuous variables and as frequency (%) for categorical variables.

Multiple imputation was conducted to address any missing data. All the clinical and MRI variables were included in the model as independent variables (predictor). Incidence of (2 months and 12 months) reinjury was set as a dependent variable. The Markov Chain Monte Carlo method was used to impute 873 (5.27%) missing values. 200 repeating procedures were performed, and the fully conditional specification method fits a univariate model using all other available variables in the model as predictors, then imputed missing values for the variable being fit. The method continued until the maximum number of iterations was reached. A pooled dataset was used for analysis.

The linearity assumption in logistic regression was conducted to assess the linear relationship between the quantitative predictor variables and the outcome (reinjury). For the univariate analysis, a modified Poisson regression was used on the pooled dataset to investigate the association between possible predictive baseline variables and hamstring reinjury at 2 months and 12 months RTP. Variables that had a pooled p<0.1 on univariate testing were included in a multivariable analysis.

For the multivariable analysis, the modified Poisson regression was conducted to the included variables from previous univariate analysis (p<0.1). We also included the treatment variables of each of the studies (platelet-rich plasma/platelet-poor plasma injection received and type of rehabilitation received) in the analysis to adjust for potential confounding. We calculated the adjusted risk ratio (ARR) and 95% CI. Variables with a p<0.05 were considered independent reinjury risk factors.

## Results

### Study participants and follow-up

**Figure 1:**
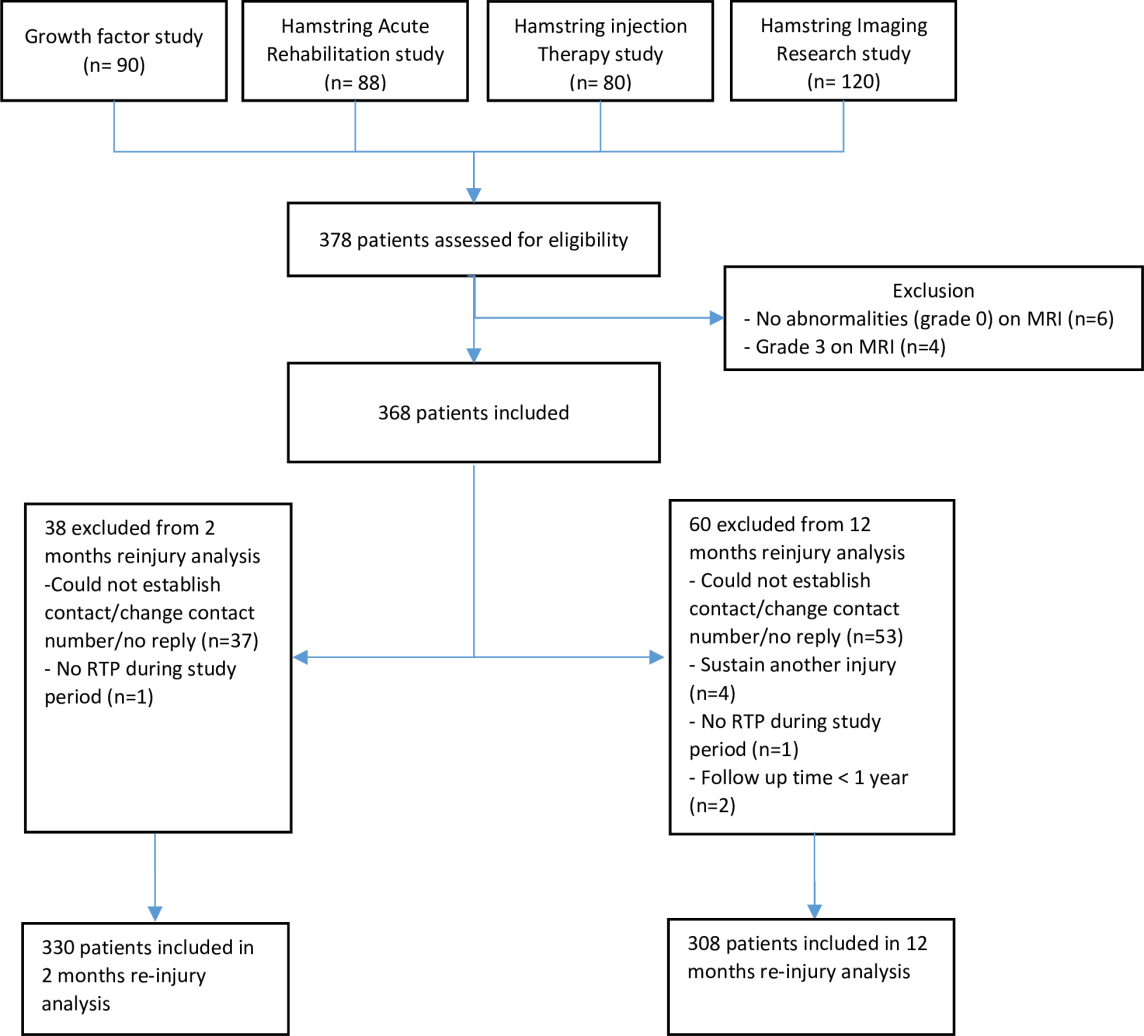
Flow diagram merging of databases and loss to follow-up. RTP, return to play.

A total of 378 patients from the Growth Factor (n=90), Rehabilitation of Acute Hamstring Injury (n=88), Hamstring Injection Therapy (n=80) and DTI for Hamstring Injury (n=120) studies were assessed for eligibility. 10 patients were excluded from the analysis: 6 patients had no abnormalities on MRI, and 4 patients had a complete proximal tendon avulsion (grade 3). Of the 368 patients, we excluded patients who had missing data regarding reinjury (n=38 for <2 months, and n=60 for <12 months) for the final analysis, resulting in 330 patients who were included in the 2-month reinjury analysis and 308 patients who were included in the 12-month reinjury analysis (figure 1). During the 2-month follow-up, a total of 31 (9%) reinjuries occurred; 52 (17%) of the reinjuries occurred within 12 months following RTP. A detailed description of characteristics is presented in online supplemental appendix 4.

### Association of clinical and MRI assessment with hamstring reinjury at 2 months following RTP

The association of baseline assessment with hamstring reinjury analysed with univariate modified Poisson regression analysis at 2 months following RTP is presented in table 1. Five variables with a p<0.1 were included in the multivariable modified Poisson regression analysis: the presence of discomfort during active knee extension test, straight leg test angle on the injured leg, the presence of MTJ involvement, extent of haematoma transverse and extent of haematoma craniocaudal. One finding was independently associated with reinjury risk (table 2): the presence of discomfort during active knee extension test on the injured leg (ARR 3.38; 95% CI 1.185 to 9.641; p=0.023).

**Table 1:** Univariate results of the association between the clinical and MRI findings at initial injury and event of reinjury at 2 months (n=31; 9%) follow-up

Variable	No reinjury (n=299)	Reinjury (n=31; 9%)	ARR (95% CI)	P value	Trend of prognosis of reinjury
**Patient characteristics**					
Categorical variables* (%)					
Previous hamstring injury					
No	48.00%	37.80%	1.473 (0.695 to 3.121)	0.312	↑
Yes	52.00%	62.20%			
Previous ipsilateral hamstring injury					
No	59.00%	45.90%	1.598 (0.792 to 3.284)	0.187	↑
Yes	41.00%	54.10%			
Level of sport					
Recreational	7.70%	13.20%	0.593 (0.224 to 1.570)	0.293	↓
Competitive/professional	92.30%	86.80%			
Continuous variables†, mean (SD)					
Age (years)	26.7 (7.2)	27.0 (6.3)	1.005 (0.966 to 1.046)	0.813	↑
Height (cm)	179 (8)	180 (8)	1.005 (0.963 to 1.050)	0.819	↑
Weight (kg)	77.0 (11.4)	76.8 (8.8)	0.999 (0.974 to 0.976)	0.919	↓
BMI (kg/m^2^)	23.9 (2.6)	23.8 (7.3)	0.978 (0.891 to 0.931)	0.644	↓
**Clinical variables**					
Categorical variables* (%)					
Sprinting injury type					
Sprinting	27.60%	32.30%	1.220 (0.598 to 2.492)	0.585	↑
No sprinting	72.40%	67.70%			
Discomfort restricted flexion 90^o^					
Discomfort/pain present	82.60%	80.60%	0.887 (0.379 to 2.075)	0.783	↓
No discomfort/pain	17.40%	19.40%			
Discomfort during active knee extension					
Discomfort/pain present	57.90%	67.80%	2.646 (1.231 to 5.686)	0.013	↑
No discomfort/pain	42.00%	32.20%			
Continuous variables†, mean (SD)					
Time to RTP (days)	40 (31.2)	34.3 (18.5)	0.992 (0.979 to 1.005)	0.235	↓
Length of painful area during palpation (cm)	10.2 (10.2)	8.9 (7.2)	0.986 (0.948 to 0.974)	0.494	↓
Straight leg raise angle					
Injured leg (degrees)	70.5 (17.5)	63.8 (17.6)	0.981 (0.965 to 0.998)	0.029	↓
Active knee extension angle					
Injured leg (degrees)	72.8 (39.1)	82.0 (40.6)	1.005 (0.997 to 1.014)	0.235	↑
Uninjured leg (degrees)	92.6 (32.4)	96.6 (40.6)	1.003 (0.992 to 1.015)	0.589	↑
Deficit (degrees)	19.9 (23.1)	14.6 (19.5)	0.990 (0.975 to 1.015)	0.193	↓
Isometric knee flexion force in 15°					
Injured leg (Newton)	146.2 (77.2)	139.1 (75.8)	0.999 (0.994 to 1.003)	0.631	↓
Uninjured leg (Newton)	248.5 (52.9)	236.7(65.8)	0.997 (0.992 to 1.003)	0.335	↓
Deficit (Newton)	102.3 (75.2)	97.5 (77.2)	0.999 (0.995 to 1.004)	0.747	↓
Isometric knee flexion force in 90°					
Injured leg (Newton)	134.6 (63.2)	148.2 (57.0)	1.004 (0.998 to 1.010)	0.154	↑
Uninjured leg (Newton)	176.7 (47.8)	185.7 (52.7)	1.004 (0.996 to 1.011)	0.363	↑
Deficit (Newton)	42.1 (54.1)	37.5 (49.8)	0.999 (0.993 to 1.005)	0.635	↓
**MRI variables**					
Categorical variables* (%)					
Involved muscles					
Biceps femoris	77.30%	77.40%	1.008 (0.452 to 2.247)	0.984	↑
Semimembranosus/semitendinosus	22.70%	22.60%			
Modified Peetrons grading on MRI					
Grade 1	33.40%	32.30%	1.050 (0.512 to 2.151)	0.894	↑
Grade 2	66.60%	67.70%			
Tendon involvement					
No tendon involvement	46.80%	45.20%	1.063 (0.542 to 2.083)	0.86	↑
Tendon involvement	53.20%	54.80%			
MTJ involvement					
No MTJ involvement	30.10%	12.90%	2.689 (0.968 to 7.478)	0.058	↑
MTJ involvement	69.90%	87.10%			
IM tendon disruption					
No IM tendon disruption	49.80%	43.40%	0.794 (0.399 to 1.578)	0.509	↓
IM tendon disruption	50.20%	56.60%			
Complete IM tendon disruption					
No complete disruption	90.60%	95.70%	0.433 (0.066 to 2.838)	0.383	↓
Complete disruption	9.40%	4.30%			
Presence of waviness					
No waviness	62.10%	62.40%	0.988 (0.488 to 2.000)	0.973	↓
Waviness present	37.90%	37.60%			
Presence of fibrosis					
No fibrosis	92.00%	86.60%	1.664 (0.627 to 4.415)	0.307	↑
Fibrosis present	8.00%	13.40%			
Continuous variables†, mean (SD)					
Extent of oedema anteroposterior (cm)	2.2 (1.3)	2.1 (1.1)	0.955 (0.751 to 1.214)	0.706	↓
Extent of oedema transverse (cm)	2.4 (1.2)	2.1 (1.2)	0.827 (0.592 to 1.155)	0.265	↓
Extent of oedema craniocaudal (cm)	14.0 (7.6)	12.8 (6.2)	0.981 (0.940 to 1.271)	0.379	↓
Extent of haematoma anteroposterior (cm)	0.4 (0.6)	0.5 (0.5)	1.319 (0.871 to 1.998)	0.19	↑
Extent of haematoma transverse (cm)	0.4 (0.6)	0.6 (0.8)	1.445 (0.978 to 2.132)	0.064	↑
Extent of haematoma craniocaudal (cm)	1.5 (2.7)	2.4 (4.1)	1.067 (0.995 to 1.145)	0.067	↑
Length of IM tendon disruption (cm)	3.1 (4.5)	3.5 (4.4)	1.012 (0.946 to 1.083)	0.717	↑

↑=trending towards a positive association/increased with reinjury risk (ARR higher than 1); ↓=trending towards a negative association/decreased with reinjury risk (ARR less than 1).

*Categorical variable data given as pooled percentages from 200 multiple-imputation variations.

†Continuous variables data given as pooled mean from 200 multiple-imputation variations.

ARR, adjusted risk ratio; BMI, body mass index; IM, intramuscular; MTJ, myotendinous junction; RTP, return to play.

**Table 2:** Multivariable results of the association between the clinical and MRI findings at initial injury and event of 2 months (31 (9%) reinjuries) and 12 months reinjuries (52 (17%) reinjuries)

Variable	ARR (95% CI)	P value	Trend of prognosis of reinjury
2 months hamstring reinjury			
Clinical findings			
Straight leg raise angle on injured leg	0.972 (0.945 to 1.000)	0.054	↓
Presence of discomfort during active knee extension test	3.380 (1.185 to 9.641)	0.023	↑
MRI findings			
MTJ involvement	2.522 (0.804 to 7.909)	0.113	↑
Extent of haematoma transverse	1.200 (0.715 to 2.012)	0.490	↑
Extent of haematoma craniocaudal	1.004 (0.909 to 1.107)	0.940	↑
12 months hamstring reinjury			
Clinical findings			
Previous hamstring Injury	1.820 (0.624 to 5.307)	0.273	↑
Previous ipsilateral hamstring injury	1.359 (0.548 to 3.370)	0.508	↑
Time to RTP	0.985 (0.970 to 0.999)	0.043	↓
Injury mechanism: sprinting vs non sprinting	0.768 (0.380 to 1.550)	0.461	↓
Presence of discomfort during active knee extension test	2.517 (1.096 to 5.783)	0.030	↑
Straight leg raise angle on injured leg	0.975 (0.955 to 0.996)	0.021	↓
Active knee extension test on Injured leg	0.096 (0.981 to 1.011)	0.607	↓
Active knee extension test on uninjured leg	0.999 (0.981 to 1.017)	0.938	↓
Isometric strength test at 90^o^ on injured leg	1.002 (0.996 to 1.008)	0.527	↑
Isometric strength test at 90^o^ on injured leg uninjured leg	1.002 (0.995 to 1.009)	0.505	↑
MRI findings			
Extent of oedema anteroposterior	0.740 (0.570 to 0.961)	0.024	↓
Extent of haematoma transverse	1.445 (0.897 to 2.328)	0.131	↑
MTJ involvement	3.099 (1.387 to 6.931)	0.006	↑
Presence of waviness	0.712 (0.393 to 1.288)	0.261	↓

↑=trending towards a positive association/increased with reinjury risk (ARR higher than 1); ↓=trending towards a negative association/decreased with reinjury risk (ARR less than 1).

ARR, adjusted risk ratio; MTJ, myotendinous junction; RTP, return to play.

### Association of clinical and MRI assessment with hamstring reinjury at 12 months following RTP

The association of baseline assessment with hamstring reinjury analysed with univariate modified Poisson regression analysis at 12 months following RTP is presented in table 3. 14 variables with a p<0.1 were included in the multivariable analysis, including 10 clinical findings and 4 MRI findings. Multivariable modified Poisson regression analyses were conducted to the 14 variables, of which 5 findings were independently significant (table 2): time to RTP (ARR 0.985; 95% CI 0.970 to 0.999; p=0.043), the presence of discomfort during active knee extension test (ARR 2.517 ; 95% CI 1.096 to 5.783; p=0.030), straight leg raise angle on injured leg (ARR 0.975; 95% CI 0.95 to 0.996; p=0.021), MTJ involvement (ARR 3.099; 95% CI 1.387 to 6.931; p=0.006) and extent of oedema anteroposterior (ARR 0.740; 95% CI 0.570 to 0.961; p=0.024).

**Table 3:** Univariate results of the association between the clinical and MRI variables at initial injury and event of reinjury at 12 months (n=52; 17%) follow-up

Variable	No reinjury (n=256)	Reinjury (n=52; 17%)	ARR (95% CI)	P value	Trend of prognosis of reinjury
**Patient characteristics**					
Categorical variables* (%)					
Previous hamstring injury					
No	49.70%	33.50%	1.768 (1.016 to 3.074)	0.044	↑
Yes	50.30%	66.50%			
Previous ipsilateral hamstring injury					
No	59.90%	43.60%	1.733 (1.036 to 2.904)	0.036	↑
Yes	40.10%	56.40%			
Level of sport					
Recreational	8.60%	7.90%	1.093 (0.428 to 2.787)	0.853	↑
Competitive/professional	91.40%	92.10%			
Continuous variables†, mean (SD)					
Age (years)	26.9 (7.4)	25.6 (6.2)	0.995 (0.963 to 1.028)	0.763	↓
Height (cm)	179 (10)	180 (10)	1.012 (0.981 to 1.044)	0.46	↑
Weight (kg)	76.8 (11.1)	76.5 (10.0)	0.998 (1.011 to 1.020)	0.874	↓
BMI (kg/m^2^)	23.9 (2.6)	23.6 (2.0)	0.954 (0.870 to 1.047)	0.322	↓
**Clinical variables**					
Categorical variables* (%)					
Sprinting injury type					
Sprinting	26.40%	38.50%	1.571 (0.952 to 2.593)	0.077	↑
No sprinting	73.60%	61.50%			
Discomfort restricted flexion 90^o^					
Present	82.10%	84.50%	1.156 (0.574 to 2.328)	0.685	↑
Absent	17.90%	15.50%			
Discomfort during active knee extension					
Present	42.40%	59.30%	1.765 (1.025 to 3.040)	0.04	↑
Absent	57.60%	40.70%			
Continuous variables†, mean (SD)					
Time to RTP (days)	40.2 (32.5)	32.9 (16.6)	0.989 (0.979 to 0.999)	0.039	↓
Length of painful area during palpation (cm)	10.3 (10.5)	8.8 (6.5)	0.984 (0.957 to 1.012)	0.272	↓
Straight leg raise angle					
Injured leg (degrees)	69.9 (17.2)	65.4 (17.5)	0.988 (0.974 to 1.002)	0.088	↓
Active knee extension angle					
Injured leg (degrees)	71.9 (38.9)	83.8 (43.7)	1.006 (1.000 to 1.012)	0.059	↑
Uninjured leg (degrees)	91.5 (32.1)	101.2 (39.5)	1.007 (0.999 to 1.015)	0.088	↑
Deficit (degrees)	19.6 (23.5)	17.4 (18.9)	0.996 (0.986 to 1.006)	0.465	↓
Isometric knee flexion force in 15°					
Injured leg (Newton)	146.0 (75.9)	141.4 (79.9)	0.999 (0.996 to 1.003)	0.709	↓
Uninjured leg (Newton)	247.0 (63.3)	241.4 (64.4)	0.999 (0.995 to 1.003)	0.565	↓
Deficit (Newton)	101.0 (73.6)	100.0 (79.1)	1.000 (0.996 to 1.003)	0.931	–
Isometric knee flexion force in 90°					
Injured leg (Newton)	133.1 (52.5)	147.6 (48.2)	1.004 (1.000 to 1.009)	0.043	↑
Uninjured leg (Newton)	174.3 (46.4)	186.8 (47.5)	1.005 (0.999 to 1.010)	0.087	↑
Deficit (Newton)	41.2 (55.4)	39.1 (47.9)	0.999 (0.995 to 1.004)	0.786	↓
**MRI variables**					
Categorical variables* (%)					
Involved muscles					
Biceps Femoris	75.80%	84.60%	1.618 (0.799 to 3.274)	0.181	↑
Semimembranosus/semitendinosus	24.20%	15.40%			
Modified Peetrons grading on MRI					
Grade 1	33.20%	32.70%	1.019 (0.601 to 1.730)	0.943	↑
Grade 2	66.80%	67.30%			
Tendon involvement					
No tendon involvement	46.10%	50.00%	0.878 (0.535 to 1.441)	0.607	↓
Tendon involvement	53.90%	50.00%			
MTJ involvement					
No MTJ involvement	30.90%	15.40%	2.164 (1.063 to 4.411)	0.033	↑
MTJ involvement	69.10%	84.60%			
IM tendon disruption					
No IM tendon disruption	50.90%	54.70%	1.208 (0.731 to 1.998)	0.46	↑
IM tendon disruption	49.10%	45.30%			
Complete IM tendon disruption					
No complete disruption	91.30%	95.50%	0.531 (0.143 to 1.978)	0.345	↓
Complete disruption	8.70%	4.50%			
Presence of waviness					
No waviness	61.00%	73.70%	0.611 (0.342 to 1.091)	0.096	↓
Waviness present	40.00%	26.30%			
Presence of fibrosis					
No fibrosis	92.00%	88.20%	1.409 (0.664 to 2.989)	0.371	↑
Fibrosis present	8.00%	11.70%			
Continuous variables†, mean (SD)					
Extent of oedema anteroposterior (cm)	2.2 (1.4)	1.9 (1.1)	0.837 (0.691 to 1.013)	0.068	↓
Extent of oedema transverse (cm)	2.4 (1.3)	2.2 (1.1)	0.867 (0.706 to 1.065)	0.174	↓
Extent of oedema craniocaudal (cm)	14.0 (7.5)	12.5 (6.3)	0.976 (0.945 to 1.009)	0.154	↓
Extent of haematoma anteroposterior (cm)	0.4 (0.6)	0.5 (00.4)	1.252 (0.914 to 1.714)	0.162	↑
Extent of haematoma transverse (cm)	0.4 (0.6)	0.6 (0.7)	1.326 (1.002 to 1.754)	0.048	↑
Extent of haematoma craniocaudal (cm)	1.5 (2.8)	2.1 (3.4)	1.045 (0.988 to 1.104)	0.122	↑
Length of IM tendon disruption (cm)	3.2 (4.6)	2.8 (4.3)	0.981 (0.925 to 1.040)	0.521	↓

↑=trending towards a positive association/increased with reinjury risk (ARR higher than 1); ↓=trending towards a negative association/decreased with reinjury risk (ARR less than 1); –=trending towards an equal association with reinjury risk (ARR=1).

*Categorical variable data given as pooled percentages from 200 multiple-imputation variations.

†Continuous variables data given as pooled mean from 200 multiple-imputation variation.

ARR, adjusted risk ratio; BMI, body mass index; IM, intramuscular; MTJ, myotendinous junction; RTP, return to play.

## Discussion

This represents the largest analysis of data from prospective (merged) cohort studies with over 300 hamstring injuries and 52 reinjuries. The main findings are that for reinjuries occurring within 2 months, the presence of discomfort during the active knee extension test was independently associated with increased reinjury risk. For reinjuries occurring within 12 months, the presence of discomfort during active knee extension test, shorter time to RTP, lower straight leg raise angle on injured leg, the MTJ involvement and less extent of oedema anteroposterior on MRI was independently associated with reinjury risk.

The five findings are newly identified predictors, whereas previous hamstring injury that has previously been identified as a risk factor was not strong predictor in this analysis. As (delaying) the time to RTP and performing a baseline MRI are in the hands of the medical staff, we recommend considering the prolonged time to RTP in high-risk athletes (based on the risk profiling) and performing MRI as the preferential baseline imaging modality in the evaluation following hamstring injuries.

### Baseline clinical examination: presence of discomfort during active knee extension test and straight leg raise angle on the injured leg

The presence of discomfort during the active knee extension test was a significant risk factor both in the 2-month and 12-month follow-up. This finding had the highest predicting value (ARR 3.380; 95% CI 1.185 to 9.641) in our cohort. It is associated with a three times higher risk to sustain a reinjury within 2-month compared with patients without discomfort during active knee extension test. This is a novel finding, as none of the previous studies investigated the association of the active knee extension test with reinjury.^27–29^ In our previous substudy with a smaller sample size (Hamstring Injection Therapy Study, n=64), we reported that a flexibility deficit during the active knee extension examined just after RTP was a risk factor of 1-year reinjury.^1^ Now, in this study with a larger merged cohort,^14–16^ we identified comparable findings on the active knee extension test, indicating that the test is a clinically meaningful test to evaluate hamstring reinjury risk.

In addition to the active knee extension test, we also found that straight leg raise angle on injured leg was negatively associated with reinjury risk. A higher angle degree of straight leg raise on injured leg will decrease the reinjury relative risk by 3% (ARR 0.97). A prospective study in male soccer players reported that soccer players with increased hamstring tightness have a statistically higher risk for a subsequent muscle injury.^30^ Both active knee extension and straight leg raise tests were widely used for flexibility assessment and had excellent inter-tester reliability.^18 31^ Therefore, it should be used in the clinical toolbox to evaluate hamstring health.

### Baseline MRI: hamstring injury with MTJ involvement and extent of oedema anteroposterior

We found that MRI-detected MTJ involvement was a risk factor for 12-month reinjury, with almost three times higher risk than injury without MTJ involvement (ARR 3.099; 95% CI 1.387 to 6.931). For 2-month reinjury, MTJ also has a positive (but statistically non-significant) association with the 2-month reinjury (ARR 2.522; 95% CI 0.804 to 7.909).

MTJ involvement has not been mentioned in previous studies on hamstring reinjury risk.^32–34^ A meta-analysis reported that at both baseline and at RTP, MRI findings were not associated with a greater risk of hamstring reinjury.^9^ Two systematic review studies from de Visser *et al* 2 and van Heumen *et al* 11 mentioned other MRI findings as hamstring reinjury risk factors with limited to moderate evidence including grade 1 injury, larger volume of injury, biceps femoris muscle injury and intratendinous injury on MRI. Thus, studies investigating the association of IM tendon injury observed on MRI with reinjury risk have reported conflicting results.^35 36^

The MTJ is the interface between muscle and tendon,^37^ and it has been reported as a common location for hamstring injury in sports.^38^ Injuries in this area of the hamstring muscles occur during fast eccentric actions where the MTJ, as a ‘weak spot’, is exposed to high loads during lengthening, especially at late swing and the early stance phase during running and/or rapid change of direction.^39 40^ Standard clinical practice suggests the use of eccentric strengthening exercise (ie, Nordic Hamstring Exercise) as a way to prevent primary acute hamstring injury, possibly due to the reported effectiveness in football players.^41 42^ However, new evidence suggests that a combination of eccentric exercises may have an even better chance of protecting hamstring muscles from reinjuries.^43^ Further studies to evaluate the effectiveness of a comprehensive exercise approach to prevent hamstring reinjury need to be conducted.^44^

What we know is that inactivity or unloading can reduce the surface area of the MTJ.^40^ Theoretically, the first injury that requires a period of immobilisation might weaken the MTJ by making it less capable to tolerate load—and therefore more susceptible to injury. This may be why reinjury tends to occur early, especially in the first months after RTP.^45^ For this reason, when MRI assessments are available, clinicians should also consider assessing the integrity of the MTJ when injuries in this area occur.

In this study, we found contradictive result that extent of oedema anteroposterior has a negative association with reinjury (ARR 0.740; 95% CI 0.570 to 0.961). It means longer oedema (cm) in the baseline MRI in the anteroposterior plane may decrease the reinjury risk within 12 months. This negative association was not found for oedema measured in the craniocaudal and transverse planes. Further study needs to be conducted to confirm whether this unexpected result occurred due to random variation or indicative of a true association between variables. However, these two MRI findings might serve as a strong argument to consider MRI as the preferential imaging modality in the clinical assessment following hamstring injuries.

### Time to RTP

We found that a longer time to RTP decreased the relative risk of 12-month reinjury; specifically, reinjury risk decreased by 1.5% (ARR 0.985) per day, which means that if the patient prolongs RTP by 4 days, their absolute risk of reinjury is decreased by 6%. This mimics a recent statement that functional recovery precedes the biological healing of the muscle. We recommend that in a shared decision elite-athlete setting, medical staff should emphasise the decreased reinjury risk by prolonged RTP time.

### Strengths and limitations

The main strength of this study is the large sample size of 330 acute hamstring injuries with a subsequent high number of reinjuries, 31 cases within 2 months and 52 cases within 12 months. This prospective cohort data set provides good sensitivity to identify the association between risk factors and outcomes and helps minimise the risk of biases (ie, recall and selection bias). The clinical examination was performed with similar standardised procedures across the different study cohorts. MRIs were scored using a standardised data collection form with good interobserver and intraobserver reliability.^23 25^ We used multivariable analysis with a modified Poisson regression approach to examine the independent association between the baseline findings and reinjury. Therefore, the study might have sufficient power and robust prospective design to provide an initial attempt to report the effect size of risk factors for reinjuries in moderate to strong associations.

These analyses have some limitations. First, the baseline (clinical and MRI) examinations were performed in different study centres, potentially reducing the study’s consistency and internal validity. However, standardised assessment procedures were used, and the observers were trained to minimise the risk of examiner bias. Second, most MRIs were performed on a 1.5 Tesla scanner, except the images obtained in the DTI Hamstring Imaging study that were done with 3.0 Tesla Scanner. The different magnetic strengths of MRI might influence the sensitivity in detecting any structural damage to the tissue, resulting in different interpretations of MRI scoring by the radiologist. Third, the patients in each study project followed different rehabilitation processes, either supervised by a physiotherapist/researcher or a self-guided programme. They received various treatment programmes (injection or rehabilitation), and the clearance for RTP was finalised either by the physician in the study centre or the healthcare provider outside the study centre (club, federation headquarters, private clinic). These factors are potential confounders, but this was somewhat mitigated by adjusting for these in the multivariable analysis. The other confounding factors that were not measured in this study (ie, training load and intensity, playing position, field surface) might also limit the result. Therefore, we believe our study captures real-life situations in sporting populations, and the variation in treatments received strengthens the generalisability of the findings and ecological validity. Finally, most of the study population were male patients (98%) who participated in sport at a professional level (66%). These findings may not be generalisable to female, adolescent or non-professional athletes.

## Clinical implications

Baseline clinical and MRI findings provide valuable information to the clinician for identifying patients at increased reinjury risk. In particular, time to RTP, the presence of discomfort during active knee extension test and straight leg raise angle on the injured leg, MTJ involvement and extent of oedema anteroposterior are predictors that can assist reinjury risk management following acute hamstring injuries.

As the baseline active knee extension test and straight leg raise were part of the routine clinical examination whose results cannot be influenced, the (delaying) the time to RTP and performing a baseline MRI are also in the hands of the medical staff. We recommend considering the prolonged RTP time in high-risk athletes to reduce their risk. We also recommend performing baseline MRI following acute hamstring as these two MRI findings might serve as a strong argument to consider MRI as the preferential imaging modality in the clinical assessment following hamstring injuries.

## Conclusion

Two clinical findings (presence of discomfort during active knee extension test and lower straight leg raise angle on the injured leg) and shorter time to RTP were associated with increased risk of hamstring reinjury. For MRI findings, the involvement of MTJ and extent of oedema anteroposterior were associated with hamstring reinjury risk.

## Data Availability

All data relevant to the study are included in the article or uploaded as online supplemental information.

## References

[1] De Vos R-J , Reurink G , Goudswaard G-J , et al . Clinical findings just after return to play predict hamstring re-injury, but baseline MRI findings do not. Br J Sports Med 2014;48:1377–84. 10.1136/bjsports-2014-093737 25037201

[2] de Visser HM , Reijman M , Heijboer MP , et al . Risk factors of recurrent hamstring injuries: a systematic review. Br J Sports Med 2012;46:124–30. 10.1136/bjsports-2011-090317 22011915

[3] Edouard P , Pollock N , Guex K , et al . Hamstring muscle injuries and hamstring specific training in elite athletics (track and field) athletes. Int J Environ Res Public Health 2022;19:10992. 10.3390/ijerph191710992 36078705 PMC9518337

[4] Ekstrand J , Bengtsson H , Waldén M , et al . Hamstring injury rates have increased during recent seasons and now constitute 24% of all injuries in men’s professional football: the UEFA elite club injury study from 2001/02 to 2021/22. Br J Sports Med 2022;57:292–8. 10.1136/bjsports-2021-105407 36588400 PMC9985757

[5] Reurink G , Almusa E , Goudswaard GJ , et al . No association between fibrosis on magnetic resonance imaging at return to play and hamstring Reinjury risk. Am J Sports Med 2015;43:1228–34. 10.1177/0363546515572603 25748473

[6] Saw R , Finch CF , Samra D , et al . Injuries in Australian rules football: an overview of injury rates, patterns, and mechanisms across all levels of play. Sports Health 2018;10:208–16. 10.1177/1941738117726070 28825878 PMC5958447

[7] Orchard J , Best TM . The management of muscle strain injuries: an early return versus the risk of recurrence. Clin J Sport Med 2002;12:3–5. 10.1097/00042752-200201000-00004 11854581

[8] Al Attar WSA , Soomro N , Sinclair PJ , et al . Effect of injury prevention programs that include the Nordic hamstring exercise on hamstring injury rates in soccer players: a systematic review and meta-analysis. Sports Med 2017;47:907–16. 10.1007/s40279-016-0638-2 27752982

[9] Green B , Bourne MN , van Dyk N , et al . Recalibrating the risk of hamstring strain injury (HSI): a 2020 systematic review and meta-analysis of risk factors for index and recurrent hamstring strain injury in sport. Br J Sports Med 2020;54:1081–8. 10.1136/bjsports-2019-100983 32299793

[10] Freckleton G , Pizzari T . Risk factors for hamstring muscle strain injury in sport: a systematic review and meta-analysis. Br J Sports Med 2013;47:351–8. 10.1136/bjsports-2011-090664 22763118

[11] van Heumen M , Tol JL , de Vos R-J , et al . The prognostic value of MRI in determining reinjury risk following acute hamstring injury: a systematic review. Br J Sports Med 2017;51:1355–63. 10.1136/bjsports-2016-096790 28259847

[12] Isern-Kebschull J , Pedret C , Mechó S , et al . MRI findings prior to return to play as predictors of reinjury in professional athletes: a novel decision-making tool. Insights Imaging 2022;13:203. 10.1186/s13244-022-01341-1 36575363 PMC9794673

[13] Bahr R , Holme I . Risk factors for sports injuries--a methodological approach. Br J Sports Med 2003;37:384–92. 10.1136/bjsm.37.5.384 14514527 PMC1751357

[14] Hamilton B , Tol JL , Almusa E , et al . Platelet-rich plasma does not enhance return to play in hamstring injuries: a randomised controlled trial. Br J Sports Med 2015;49:943–50. 10.1136/bjsports-2015-094603 26136179

[15] Reurink G , Goudswaard GJ , Moen MH , et al . Platelet-rich plasma injections in acute muscle injury. N Engl J Med 2014;370:2546–7. 10.1056/NEJMc1402340 24963588

[16] Vermeulen R , Whiteley R , van der Made AD , et al . Early versus delayed lengthening exercises for acute hamstring injury in male athletes: a randomised controlled clinical trial. Br J Sports Med 2022;56:792–800. 10.1136/bjsports-2020-103405 35338036 PMC9252858

[17] Wangensteen A , Almusa E , Boukarroum S , et al . MRI does not add value over and above patient history and clinical examination in predicting time to return to sport after acute hamstring injuries: a prospective cohort of 180 male athletes. Br J Sports Med 2015;49:1579–87. 10.1136/bjsports-2015-094892 26305004

[18] Reurink G , Goudswaard GJ , Oomen HG , et al . Reliability of the active and passive knee extension test in acute hamstring injuries. Am J Sports Med 2013;41:1757–61. 10.1177/0363546513490650 23735425

[19] Landes S , Nyland J , Elmlinger B , et al . Knee flexor strength after ACL reconstruction: comparison between hamstring autograft, tibialis anterior allograft, and non-injured controls. Knee Surg Sports Traumatol Arthrosc 2010;18:317–24. 10.1007/s00167-009-0931-9 19898836

[20] Askling CM , Tengvar M , Saartok T , et al . Acute first-time hamstring strains during high-speed running: a longitudinal study including clinical and magnetic resonance imaging findings. Am J Sports Med 2007;35:197–206. 10.1177/0363546506294679 17170160

[21] Monte JR , Hooijmans MT , Froeling M , et al . The repeatability of bilateral diffusion Tensor imaging (DTI) in the upper leg muscles of healthy adults. Eur Radiol 2020;30:1709–18. 10.1007/s00330-019-06403-5 31705253 PMC7033061

[22] van der Made AD , Almusa E , Whiteley R , et al . Intramuscular tendon involvement on MRI has limited value for predicting time to return to play following acute hamstring injury. Br J Sports Med 2018;52:83–8. 10.1136/bjsports-2017-097659 28903949

[23] Hamilton B , Whiteley R , Almusa E , et al . Excellent reliability for MRI grading and Prognostic parameters in acute hamstring injuries. Br J Sports Med 2014;48:1385–7. 10.1136/bjsports-2013-092564 24037670 PMC4174178

[24] Reurink G , Goudswaard GJ , Tol JL , et al . MRI observations at return to play of clinically recovered hamstring injuries. Br J Sports Med 2014;48:1370–6. 10.1136/bjsports-2013-092450 24255767 PMC4174122

[25] Wangensteen A , Tol JL , Roemer FW , et al . Intra- and Interrater reliability of three different MRI grading and classification systems after acute hamstring injuries. Eur J Radiol 2017;89:182–90. 10.1016/j.ejrad.2017.02.010 28267537

[26] Reurink G , Goudswaard GJ , Moen MH , et al . Rationale, secondary outcome scores and 1-year follow-up of a randomised trial of platelet-rich plasma injections in acute hamstring muscle injury: the Dutch hamstring injection therapy study. Br J Sports Med 2015;49:1206–12. 10.1136/bjsports-2014-094250 25940636

[27] Gabbe BJ , Bennell KL , Finch CF , et al . Predictors of hamstring injury at the elite level of Australian football. Scand J Med Sci Sports 2006;16:7–13. 10.1111/j.1600-0838.2005.00441.x 16430675

[28] Rolls A , George K . The relationship between hamstring muscle injuries and hamstring muscle length in young elite footballers. Physical Therapy in Sport 2004;5:179–87. 10.1016/j.ptsp.2004.08.005

[29] Schuermans J , Van Tiggelen D , Witvrouw E . Prone hip extension muscle recruitment is associated with hamstring injury risk in amateur soccer. Int J Sports Med 2017;38:696–706. 10.1055/s-0043-103016 28704884

[30] Witvrouw E , Danneels L , Asselman P , et al . Muscle flexibility as a risk factor for developing muscle injuries in male professional soccer players. A prospective study. Am J Sports Med 2003;31:41–6. 10.1177/03635465030310011801 12531755

[31] Neto T , Jacobsohn L , Carita AI , et al . Reliability of the active-knee-extension and straight-leg-raise tests in subjects with flexibility deficits. J Sport Rehabil 2015;24:2014-0220. 10.1123/jsr.2014-0220 25364856

[32] Verrall GM , Slavotinek JP , Barnes PG , et al . Assessment of physical examination and magnetic resonance imaging findings of hamstring injury as predictors for recurrent injury. J Orthop Sports Phys Ther 2006;36:215–24. 10.2519/jospt.2006.36.4.215 16676871

[33] Gibbs NJ , Cross TM , Cameron M , et al . The accuracy of MRI in predicting recovery and recurrence of acute grade one hamstring muscle strains within the same season in Australian rules football players. J Sci Med Sport 2004;7:248–58. 10.1016/s1440-2440(04)80016-1 15362322

[34] Koulouris G , Connell DA , Brukner P , et al . Magnetic resonance imaging parameters for assessing risk of recurrent hamstring injuries in elite athletes. Am J Sports Med 2007;35:1500–6. 10.1177/0363546507301258 17426283

[35] van der Made AD , Almusa E , Reurink G , et al . Intramuscular tendon injury is not associated with an increased hamstring reinjury rate within 12 months after return to play. Br J Sports Med 2018;52:1261–6. 10.1136/bjsports-2017-098725 29654058

[36] Pollock N , Patel A , Chakraverty J , et al . Time to return to full training is delayed and recurrence rate is higher in Intratendinous ('C’) acute hamstring injury in elite track and field athletes: clinical application of the British athletics muscle injury classification. Br J Sports Med 2016;50:305–10. 10.1136/bjsports-2015-094657 26888072

[37] Huijing PA . Muscle as a collagen fiber reinforced composite: a review of force transmission in muscle and whole limb. J Biomech 1999;32:329–45. 10.1016/s0021-9290(98)00186-9 10213024

[38] De Smet AA , Best TM . MR imaging of the distribution and location of acute hamstring injuries in athletes. AJR Am J Roentgenol 2000;174:393–9. 10.2214/ajr.174.2.1740393 10658712

[39] Danielsson A , Horvath A , Senorski C , et al . The mechanism of hamstring Injuries- a systematic review. BMC Musculoskelet Disord 2020;21:641. 10.1186/s12891-020-03658-8 32993700 PMC7526261

[40] Jakobsen JR , Krogsgaard MR . The myotendinous junction-a vulnerable companion in sports. Front Physiol 2021;12:635561. 10.3389/fphys.2021.635561 33841171 PMC8032995

[41] Ekstrand J , Bengtsson H , Walden M , et al . Still poorly adopted in male professional football: but teams that used the Nordic hamstring exercise in team training had fewer hamstring injuries {\Textendash} a retrospective survey of 17 teams of the UEFA elite club injury study during the 2020{\Texte. BMJ Open Sport Exerc Med 2022;8:e001368. 10.1136/bmjsem-2022-001368 PMC9315904 35979432

[42] Arnason A , Andersen TE , Holme I , et al . Prevention of hamstring strains in elite soccer: an intervention study. Scandinavian Med Sci Sports 2008;18:40–8. 10.1111/j.1600-0838.2006.00634.x 17355322

[43] Suskens JJM , Secondulfo L , Kiliç Ö , et al . Effect of two eccentric hamstring exercises on muscle architectural characteristics assessed with diffusion tensor MRI. Scand J Med Sci Sports 2023;33:393–406. 10.1111/sms.14283 36514886

[44] Zein MI , Reurink G , Verhagen E , et al . Study on hamstring re-injury prevention (SHARP): protocol for an international multicentre, randomised controlled trial. BMJ Open 2022;12:e065816. 10.1136/bmjopen-2022-065816 PMC9664273 36375976

[45] Wangensteen A , Tol JL , Witvrouw E , et al . Hamstring reinjuries occur at the same location and early after return to sport: a descriptive study of MRI-confirmed reinjuries. Am J Sports Med 2016;44:2112–21. 10.1177/0363546516646086 27184543

