# Supplementary material for: Baseline clinical and MRI risk factors for hamstring reinjury showing the value of performing baseline MRI and delaying return to play: a multicentre, prospective cohort of 330 acute hamstring injuries
Source: Br J Sports Med. 2024 May 10;58(14):766–76. doi: 10.1136/bjsports-2023-107878 (PMC11228232; doi:10.1136/bjsports-2023-107878)
Supplement: Supplementary data [file bjsports-2023-107878supp001.pdf]

Supplementary Appendix

Supplement to:

Baseline clinical and MRI risk factors for hamstring reinjury showing the value of performing baseline MRI and delaying return to play: a multicentre, prospective cohort of 330 acute hamstring injuries

Muhammad Ikhwan Zein, MD, Milo J.K. Mokkenstorm, MD, Prof. Marco Cardinale, Louis Holzhausen, PhD., Rodney Whiteley, PT, PhD, Maarten H. Moen, MD, PhD, Gustaaf Reurink, MD, PhD, Prof. Johannes L.Tol. ; for the Qatari and Dutch Hamstring Study Group.

Table of contents

**Content**	**Page**
Full authors affiliations	3
Hamstring Study Group (Qatari and Dutch)	5
Supplement Appendix 1: Method of included study	10
Supplement Appendix 2: Rehabilitation program of included study	11
Supplement Appendix 3: Data cleaning protocol	15
Supplement Appendix 4: Descriptive baseline statistics for total study population and separated into those who did or did not incur a reinjury within 2- and 12 months return to play.	17
References	22

Full authors affiliations

Muhammad Ikhwan Zein., M.D.

- Department of Orthopedic Surgery and Sports Medicine, Amsterdam UMC location University of Amsterdam, Amsterdam, The Netherlands.
- Academic Center for Evidence Based Sports Medicine (ACES), Amsterdam, The Netherlands.
- AMC/VUmc IOC Research Center, Amsterdam Collaboration on Health and Safety in Sports (ACHSS), Amsterdam, The Netherlands.
- Amsterdam Movement Sciences, Sports, Amsterdam, The Netherlands.
- Faculty of Sports Sciences, Universitas Negeri Yogyakarta, Indonesia.

Milo J.K. Mokkenstorm., M.D.

- Department of Orthopedic Surgery and Sports Medicine, Amsterdam UMC location University of Amsterdam, Amsterdam, The Netherlands.
- Academic Center for Evidence Based Sports Medicine (ACES), Amsterdam, The Netherlands.
- AMC/VUmc IOC Research Center, Amsterdam Collaboration on Health and Safety in Sports (ACHSS), Amsterdam, The Netherlands.
- Amsterdam Movement Sciences, Sports, Amsterdam, The Netherlands.

Prof. Marco Cardinale., M.D.

- Aspetar, Orthopedic and Sports Medicine Hospital, FIFA Medical Center of Excellence, Doha, Qatar.

Louis Holzhausen., Ph.D.

- Aspetar, Orthopedic and Sports Medicine Hospital, FIFA Medical Center of Excellence, Doha, Qatar.

Rodney Whiteley., P.T,, Ph.D.

- Aspetar, Orthopedic and Sports Medicine Hospital, FIFA Medical Center of Excellence, Doha, Qatar.

Maarten H. Moen., M.D., Ph.D.

- Department of Sports Medicine, Bergman Clinics, Naarden, the Netherlands.

Gustaaf Reurink., M.D., Ph.D.

- Department of Orthopedic Surgery and Sports Medicine, Amsterdam UMC location University of Amsterdam, Amsterdam, The Netherlands.
- Academic Center for Evidence Based Sports Medicine (ACES), Amsterdam, The Netherlands.
- AMC/VUmc IOC Research Center, Amsterdam Collaboration on Health and Safety in Sports (ACHSS), Amsterdam, The Netherlands.
- Amsterdam Movement Sciences, Sports, Amsterdam, The Netherlands.

Prof. Johannes L.Tol.

- Department of Orthopedic Surgery and Sports Medicine, Amsterdam UMC location University of Amsterdam, Amsterdam, The Netherlands.
- Academic Center for Evidence Based Sports Medicine (ACES), Amsterdam, The Netherlands.
- AMC/VUmc IOC Research Center, Amsterdam Collaboration on Health and Safety in Sports (ACHSS), Amsterdam, The Netherlands.
- Amsterdam Movement Sciences, Sports, Amsterdam, The Netherlands.
- Aspetar, Orthopedic and Sports Medicine Hospital, FIFA Medical Center of Excellence, Doha, Qatar.

Zein MI, *et al. Br J Sports Med* 2024; 58:1–11. doi: 10.1136/bjsports-2023-107878

Qatari and Dutch Hamstring Study Group

GF **Growth Factor study**
HIT **Hamstring Injection Therapy Study**
HAR **Rehabilitation of Acute Hamstring Injury Study**
HIR **Diffusion Tensor Imaging (DTI) for hamstring injury Study**

**Last name**	**First name and Middle Initial (s)**	**Suffix**	**Academic degrees**	**Institution**	**Location (city, state/province, country)**	**Role of contribution e.g., chair, principal investigator**	**Study Group**
Almusa	Emad		MD, PhD	Department of Radiology, Aspetar Orthopaedic and Sports Medicine Hospital	Doha, Qatar	Member of Study Group	HAR &GF
Bahr	Roald		Professor	Department of Sports Medicine at the Norwegian School of Sport Sciences, Oslo Sports Trauma Research Center	Oslo, Norway	Member of Study Group	HAR
Bierma-Zeinstra	Sita M.A		Professor	Department of Orthopedic Surgery and Sports Medicine, Erasmus University Medical Center	Rotterdam, The Netherlands	Member of Study Group	HIT
Boukarroum	Sirine		SRN	Department of Sports Medicine, Aspetar Orthopaedic and Sports Medicine Hospital	Doha, Qatar	Member of Study Group	GF

Buckens	Stan		MD., PhD	Department of Radiology and Nuclear Medicine, Radboud University Medical Center	Nijmegen, The Netherlands	Member of Study Group	HIR
Chalabi	Hakim		MD, PhD	International Medical Affairs and Programs & Centers of Excellence, Aspetar, Orthopedic and Sports Medicine Hospital	Doha, Qatar	Member of Study Group	GF
Eirale	Cristiano		PhD	Paris Saint Germain Football Club, 78100 Saint Germain en Laye	Paris, France	Member of Study Group	GF
Farooq	Abdulaziz		MPH, MSc	Deparment of Research, Aspetar, Orthopedic and Sports Medicine Hospital	Doha, Qatar	Member of Study Group	HAR & GF
Froeling	Martijn		MD, PhD	Department of Radiology, University Medical Center Utrecht	Utrecht, The Netherlands	Member of Study Group	HIR
Geertsema	Celeste		MD	Department of Sports Medicine, Aspetar Orthopaedic and Sports Medicine Hospital	Doha, Qatar	Member of Study Group	HAR
Goudswaard	Gert Jan		MD	Medical Director, Aspetar Orthopaedic and Sports Medicine Hospital	Doha, Qatar	Member of Study Group	HIT
Hamilton	Bruce		MD, PhD	High Performance Sport NZ, Millennium Institute of Sport and Health	Auckland, New Zealand	Member of Study Group	GF

Hooijmans	Melissa		PhD	Department of Biomedical Engineering and Physics, Amsterdam UMC, University of Amsterdam, Amsterdam Movement Sciences	Amsterdam, the Netherlands	Member of Study Group	HIR
Maas	Mario		Professor	Department of Radiology and Nuclear Medicine, Amsterdam UMC, University of Amsterdam, Amsterdam Movement Sciences	Amsterdam, the Netherlands	Principal Investigator of HIR study	HIT & HIR
Monte	Jithsa R.		MD	Department of Radiology and Nuclear Medicine, Amsterdam UMC, University of Amsterdam, Amsterdam Movement Sciences	Amsterdam, the Netherlands	Member of Study Group	HIR
Nederveen	Aart J		Professor	Department of Radiology and Nuclear Medicine, Amsterdam UMC, University of Amsterdam, Amsterdam Movement Sciences	Amsterdam, the Netherlands	Principal Investigator	HIR
Oudeman	Jos		MD, PhD	Department of Orthopedics, University Medical Center Utrecht	Utrecht, The Netherlands	Member of Study Group	HIR

Smithuis	Frank F.		MD., PhD	Department of Radiology and Nuclear Medicine, Amsterdam UMC, University of Amsterdam, Amsterdam Movement Sciences	Amsterdam, the Netherlands	Member of Study Group	HIR
Strijkers	Gustav J.		PhD	Department of Biomedical Engineering and Physics, Amsterdam UMC, University of Amsterdam, Amsterdam Movement Sciences	Amsterdam, the Netherlands	Member of Study Group	HIR
Suskens	Jozef J. M.		MSc	Department of Orthopedic and Sports Medicine, Amsterdam UMC, University of Amsterdam, Amsterdam Movement Sciences	Amsterdam, the Netherlands	Member of Study Group	HIR
Targett	Stephen		MD	Department of Sports Medicine, Aspetar Orthopaedic and Sports Medicine Hospital	Doha, Qatar	member of Study Group	HAR
van der Made	Anne D		MD	Department of Orthopedic and Sports Medicine, Amsterdam UMC, University of Amsterdam, Amsterdam Movement Sciences	Amsterdam, the Netherlands	Member of Study Group	HAR
van Dyk	Nicol		PT, PhD	High Performance Unit, Irish Rugby Football Union (IRFU), Dublin, Ireland	Dublin, Ireland	Member of Study Group	HAR

				Section Sports Medicine, Faculty of Health Sciences, University of Pretoria, Pretoria, South Africa<br><br>School of Public Health, Physioetherapy and Sport Science, University College Dublin, Dublin, Ireland			
Verhaar	Jan A.N.		Professor	Department of Orthopedic Surgery and Sports Medicine, Erasmus University Medical Center	Rotterdam, The Netherlands	Member of Study Group	HIT
Vermeulen	Robin		MD	Department of Orthopedic and Sports Medicine, Amsterdam UMC, University of Amsterdam, Amsterdam Movement Sciences	Amsterdam, the Netherlands	Coordinating investigator	HAR
Wangensteen	Arnlaug		PhD	Department of Sports Medicine at the Norwegian School of Sport Sciences, Oslo Sports Trauma Research Center	Oslo, Norway	Member of Study Group	HAR
Weir	Adam		MD, PhD	Department of Orthopedic Surgery and Sports Medicine, Erasmus University Medical Center	Rotterdam, The Netherlands	Member of Study Group	HIT

Supplement Appendix 1: Summary of methods of the included study.

	Growth Factor study[1]	*Rehabilitation of Acute Hamstring Injury study*[2]	*Hamstring Injection Therapy study*[3]	*DTI for Hamstring Injury study* (ongoing)
Study design	Randomized controlled trial	Randomized controlled trial	Randomized controlled trial	Prospective cohort
Country	Qatar (Aspetar), Doha	Qatar (Aspetar), Doha	Netherlands (Multicentre)	Netherlands (AUMC), Amsterdam
Intervention(s)	1. Platelet Rich Plasma (PRP) + Rehab program.<br>2. Platelet Poor Plasma (PPP) + Rehab program.<br>3. No injection, only rehab. Program.	1. Rehab. Program + early introduction to lengthening exercises (day one of rehabilitation).<br>2. Rehab. Program + late introduction to lengthening exercises (>70% of self-rated max. speed).	1. Two Platelet-rich plasma (PRP) injections + rehab program.<br>2. Two Isotonic saline injections (placebo) + rehab. Program.	Voluntary physical therapy program
Study objectives	Efficacy of PRP in enhancing RTP following hamstring injury	Effect of early introduction of lengthening exercises on time to return to play and reinjury risk.	Efficacy of PRP in enhancing RTP following hamstring injury	Correlation between Diffusion Tensor imaging parameters return to play and reinjury.
Patient recruitment	Recruitment through Qatar National Sports Medicine Program (NSMP)	Recruitment through Qatar National Sports Medicine Program (NSMP)	Nationwide referral program for suspected hamstring injury	Online recruitment or referral by physician /physical therapist.
Eligibility criteria	Inclusion:<br>- Age: 18-50<br>- Gender: Male<br>- Available for follow-up<br>- Acute onset of posterior thigh pain<br>- Presenting an MRI ≤ 5 days from injury<br>- MRI confirmed grade I or II hamstring lesion<br>- Able to perform five sessions of physiotherapy a week at the study location<br>Exclusion:<br>- Contra-indication MRI<br>- Reinjury or chronic hamstring injury<br>- Concurrent other injuries inhibiting rehabilitation<br>- Unwilling to comply with follow-up<br>- Needle phobia<br>- Overlying skin infection<br>- Diabetes/immunocompromised state<br>- Medication with increased bleeding risk<br>- Medical contraindication to injection	Inclusion:<br>- Age: 18-50<br>- Gender: Male<br>- Available for follow-up<br>- Acute onset of posterior thight pain when training or competing.<br>- Clinical diagnosis of an acute hamstring muscle injury is defined as (a) localised pain during palpation of hamstring muscle, (b) increased pain during isometric contraction (c) Localised pain when performing a passive straight leg test.<br>- Presenting an MRI ≤ 5 days from injury<br>- MRI confirmed grade I or II hamstring lesion<br>- Able to perform ≥ 3 sessions of physiotherapy a week at study location<br>Exclusion:<br>- Verified or suspected previous hamstring injury in the same leg ≤ 6 months.<br>- Chronic hamstring complaints ≥2 months<br>- Grade III injury, including complete hamstring disruption of tendon avulsion (modified Peetrons)<br>- Contraindication to MRI<br>- No intention to return to full sport activity<br>- Refusal to receive one of the two therapies.	Inclusion:<br>- Age 18-50<br>- Clinical diagnosis of an acute hamstring injury: (a) History of acute onset of posterior thigh pain, (b) localized pain on palpation, (c) localized pain on passive stretch of the hamstring, and (d) increased pain on isometric contraction<br>- Hamstring lesion on MRI (increased signal intensity on STIR and/or T2-weighted imaging)<br>Exclusion:<br>- Not capable of doing an active exercise program<br>- Previous injection therapy for this injury<br>- No intention to return to full sports activity<br>- Contraindication to MRI<br>- Refusal to receive one of the two therapies.<br>- Injury caused by extrinsic trauma<br>- Chronic low back pain<br>- Chronic hamstring complaints ≥2 months<br>- Grade III lesion (total rupture) or avulsion on MRI.	Inclusion:<br>- Age >16<br>- Clinical diagnosis of an acute hamstring injury ≤7 days defined as:<br>- Anamnestic acute injury: Acute injury, localized pain posterior thigh, pain during palpation of hamstring muscle, -passive straight leg raise, -isometric contraction.<br>Exclusion:<br>- Not capable of doing an active activity program<br>- No intention to return to full sports activity<br>- Injury caused by extrinsic trauma<br>- Contraindication to MRI<br>- Reinjury ≤2 months<br>- Complete proximal tendon avulsion (grade 3)<br>- Other concurrent injury inhibiting rehabilitation
Randomization & blinding	Three-block randomization. Blinded assessor<br>Patient blinding for injection arm.	Computer-generated, random allocation sequence.	Computer-generated permuted block scheme. All were blinded except the coordinating physician (preparation of injection).	

Supplement Appendix 2: Rehabilitation program of included study.

1. Hamstring Injection Therapy (Study Dutch Trial Register 2771)[3]

**Physiotherapist supervised program (modified Heiderscheit,2010)**[4]		
**Phase**	**Content**	**Criteria to progress**
Phase 1	**Goals:** Protection of scar formation, minimizing muscle atrophy and stimulating neuromuscular control.<br><br>**Protection**. Exercises limited to pain-free range of motion (ROM). No excessive lengthening or resistance training of the hamstrings<br><br>**Ice application**. For pain reduction, ice can be applied 2-3 times a day (maximal 3-5 minutes when using ice and maximal 15-20 minutes when using a cool pack).<br><br>**Exercises**. Ergometer cycling, low to moderate intensity stepping exercises (such as side, grapevine, and in place fast feet stepping), isometric exercises for lumbopelvic musculature, single limb balance exercises, and core-stability exercises (such as prone body bridge, side body bridge, and supine bent knee bridge). Exercises should be performed without pain.	Criteria for progression to the next phase<br><br>1. Normal walking stride without pain<br>2. very low-speed jog without pain<br>3. Pain-free isometric contraction against sub-maximal (50- 70%) resistance during prone knee flexion manual strength test in 90° knee flexion.
Phase 2	**Goals:** Regaining pain-free ROM and development of trunk and pelvis neuromuscular control with a progressive increase in movement speed.<br><br>**Protection.** No end-range lengthening of the hamstrings when muscle weakness is still present.<br><br>**Ice application.** For pain reduction, ice can be applied after exercises (maximal 3-5 minutes when using ice and maximal 15-20 minutes when using a cool pack).<br><br>**Exercises.** Gradual increase in hamstring lengthening and intensity of exercises. Agility drills and core-stability exercises are performed with a progressive increase in speed and intensity. Based on the patient's tolerance, exercises are gradually increased in hamstring lengthening. Submaximal eccentric exercises are performed near mid-hamstring length. Start with anaerobic training and sport-specific skills, but take care to avoid end-range lengthening of the hamstrings or substantial eccentric work. Running should not be performed at a speed greater than 50% of the maximal speed.	Criteria for progression to the next phase<br><br>**1.** Pain-free full strength (5/5) during prone knee flexion manual strength test in 90° knee flexion<br>**2.** Pain-free forward and backward jogging at 50% of maximum speed.
Phase 3	**Goals**: Symptom-free during all activities, normal concentric and eccentric hamstring strength through full ROM and full speeds, improvement neuromuscular control of trunk and pelvis, and improvement control in sport-specific movements.<br><br>**Protection**. ROM is unrestricted. Sprinting and explosive acceleration should be avoided until full ROM and functional movement patterns (such as running, jumping, and cutting) can be performed pain-free.<br><br>**Ice application**. For pain reduction, ice can be applied after exercises (maximal 3-5 minutes when using ice and maximal 15-20 minutes when using a cool pack).<br><br>**Exercises**. More challenging core-stability exercises by incorporating asymmetrical postures and motion exercises.	Criteria for clearance to return to play<br><br>Symptom-free (e.g., pain and stiffness) during:<br>1. full ROM;<br>2. full-speed sprinting;<br>3. sport-specific movements (such as jumping and cutting).

11

	Eccentric exercises toward the end range of motion and increasing resistance (e.g., lunche walk with trunk rotation, supine single limb chair-bridge). Agility and sport-specific drills involving quick direction changes and technique training.	
**Home exercise program (Progressive agility and trunk stabilisation from Sherry and Best)**[5]		
Phase 1	▪ Low- to moderate-intensity sidestepping, 3 × 1 minute;<br>▪ Low- to moderate-intensity grapevine stepping (lateral stepping with the trail leg going over the lead leg and then under the lead leg), both directions, 3 × 1 minute;<br>▪ Low- to moderate-intensity steps forward and backward over a tape line while moving sideways, 2 × 1 minute;<br>▪ Single-leg stand progressing from eyes open to eyes closed, 4 × 20 seconds;<br>▪ Prone abdominal body bridge (performed by using abdominal and hip muscles to hold the body in a face-down straight-plank position with the elbows and feet as the only point of contact), 4 × 20 seconds;<br>▪ Supine extension bridge (performed by using abdominal and hip muscles to hold the body in a supine hook lying position with the head, upper back, arms, and feet as the points of contact), 4 × 20 seconds;<br>▪ Side bridge, 4 × 20 seconds on each side;<br>▪ Ice while sitting for 20 minutes.	Criteria for progression to the next phase:<br><br>1. Able to walk pain-free with normal gait pattern (e.g., same stride length and stance time on the injured leg and stance leg;<br>2. Able to do a pain-free high knee march.
Phase 2	▪ Moderate- to high-intensity sidestepping, 3 × 1 minute;<br>▪ Moderate- to high-intensity grapevine stepping, 3 × 1 minute;<br>▪ Moderate- to high-intensity steps forward and backward while moving sideways, 2 × 1 minute;<br>▪ Single-leg stand windmill touches, 4 × 20 seconds of repetitive alternate hand touches;<br>▪ Push-up stabilization with trunk rotation (performed by starting at the top of a full push-up, then maintaining this position with one hand while rotating the chest toward the side of the hand that is being lifted to point toward the ceiling, pause and return to the starting position), 2 × 15 repetitions on each side;<br>▪ Fast feet on the spot (performed by jogging in place with increasing velocity, picking the foot only a few inches off the ground), 4 × 20 seconds;<br>▪ Proprioceptive neuromuscular facilitation trunk pull-downs with Thera-Band, 2 × 15 to the right and left;<br>▪ Symptom-free practice without high-speed maneuvers;<br>▪ Ice for 20 min if any symptoms of local fatigue or discomfort are present.	
Notification	- The intensity of each exercise should be such that the patient can perform the exercise pain-free;<br>- Low intensity: a velocity of movement that is less than or near that of normal walking;<br>- Moderate intensity, a velocity of movement greater than normal walking but not as great assport;<br>- High intensity, a velocity of movement similar to sport activity.	

Zein MI, *et al. Br J Sports Med* 2024; 58:1–11. doi: 10.1136/bjsports-2023-107878

2. Growth Factor study (Clinicaltrials.gov NCT 01812564)[1]

Stage	Content	Criteria to progress
Stage 1	All activities to be pain-free<br>▪ Two-leg squat, or if able, single-leg squat<br>▪ Maintain pelvis control, hip and knee alignment, squat to 45°, hold, return to start<br>▪ Supine Bridge—2 leg<br>▪ 2 s up, 2 s down (4 s total per rep.) Begin at 45°. Must reach knee-hip-shoulder in alignment. 4×15<br>▪ supine isometric heel digs<br>▪ In supine, painlessly pull heel into bed through range. Can bias with tibial IR/ER when painless.<br>▪ Exercise bike<br>▪ Upright or recumbent can be substituted with the elliptical trainer<br>▪ Isometric manual-resisted hamstring<br>▪ The therapist applied resistance isometrically in varying angles in prone<br>▪ Soft tissue massage<br>▪ Proximal and distal to the injury site, lymphatic drainage.<br>▪ Active range of motion exercises<br>▪ Supine active knee flexion and extension, then Prone active flexion and extension	Criteria to progress to stage 2:<br>1. Painless single-leg squat<br>2. Painless bike, 150W, 5 min<br>3. Full knee extension supine
Stage 2	Any exercise from stage 1 permitted, additionally:<br>▪ Supine bridge—1 leg<br>▪ Same rate as for two legs, another knee in full extension, and thighs parallel throughout the exercise. 4×15<br>▪ Walk-Jog<br>▪ Walk 20 m corners, jog the 30 m straight, painless. Begin at 25% (self-rated) jog, progress to max70%.<br>▪ Triple extension walks<br>▪ 100 m laps, every third step triple extension—i.e., alternating legs.<br>▪ 'A' drill<br>▪ Walking late swing knee extension, painless. Alternating legs, 100 m lap.<br>▪ Soft tissue massage<br>▪ Can massage the injured area. Maximum allowed pain VAS: 4/10. The therapist uses caution with any report of discomfort, monitors symptoms, and adjusts accordingly.<br>▪ Stretching<br>▪ Hamstring (supine, 90° hip flexion, knee extension); SLR (supine to the onset of discomfort, add ankle DF)<br>▪ Initially active, patient-controlled, progress to passive, end range. SLR mobilisation if indicated.<br>▪ Resisted hamstring<br>▪ Note tibial rotation as indicated. 4×15 repetitions, aiming for fatigue	Criteria to progress to stage 3:<br>1. Run ≥70% patient rated<br>2. ROM hamstrings ≥75% uninvolved side<br>3. ROM SLR ≥75% uninvolved side
Stage 3	Any exercises from stages 1 and 2, additionally:<br>▪ Single leg bridge<br>▪ 1s repetition, 2s recovery. 4×8 repetitions.<br>▪ Single leg bridge, foot on the Swiss ball<br>▪ 1s up, 2s down. 4×8 repetitions.	Criteria to progress to stage 4 (sport-specific rehab):<br>1. 100% running speed<br>2. Painless high-speed direction changes

	▪ Interval running<br>▪ 20 m jog, 30 m run. Begin running at 70% (patient-rated), progressing by 10% steps, painlessly. At 90%, progress by 5%. Monitor performance by hand timing.<br>▪ Modified T-Drill<br>▪ Direction changing running over T-Drill course. Begin at patient rated 70%, progress as able by 10% until 90%, then by 5%. Monitor performance by hand timing.<br>▪ Eccentric exercises<br>▪ Nordic Hamstrings, manual-resisted eccentric, prone catches, Arabesque (single leg stance, trunk flexion)	
Stage 4	Any exercises from stages 1–3, additionally on-field, football-specific drills:<br>▪ Direction change drills<br>▪ With and without the ball, 40 min<br>▪ Jumping drills<br>▪ 10–15 min	Criteria to progress to stage 5 (sport-specific rehab):<br>1. Painless completion of stage 4
Stage 5	Passes and run<br>Long passes progression Crosses (static)<br>Corner kicks Crosses (dynamic)	Criteria to progress to stage 6 (sport-specific rehab):<br>1. Painless completion of stage 5
Stage 6	Passes and run<br>Shooting scenarios Competitive one versus-one drill<br>Shooting scenarios<br>Scoring scenarios	Criteria to progress to medical review for return to sport:<br>1. Painless completion of stage 6

DF, dorsiflexion; ER, external rotation; IR, internal rotation; Modified T-Drill, (always) forward running over the course of the Agility t test; ROM, range of motion; SLR, straight leg raise.

3. Rehabilitation of Acute Hamstring Injury study (Clinicaltrials.gov NCT 02104258)[2]

Both groups received a similar 6-stage standard criteria-based rehabilitation program (see rehabilitation program for Growth Factor Study). The difference between the treatment groups was the introduction of the lengthening exercise (the extender, the diver, and the slider exercise)[6,7] at different time points. In the early lengthening group, the lengthening exercise was introduced on day 1 of rehabilitation. In the delayed lengthening group, the lengthening exercises were introduced after meeting the criteria of rehabilitation program stage 3 (able to run more than 70% of maximal speed).

4. DTI for Hamstring Injury study (Dutch Trial Register 2016_033).

The patient was advised to be treated using a criteria-based rehabilitation program but on a voluntary basis. The optional rehabilitation protocol, such as Aspetar,[8,9] Sherry and Best,[5] Mendighucia[10] and Askling[7]). The rehabilitation protocols were available for patients and therapists on the study website.

Supplement Appendix 3: Data cleaning protocol.

Data cleaning Merged databases (Muscle Medics)

Based on the Quality Manual of the Department of General Practice of the Erasmus MC)

Data to be cleaned

All variables merged from different databases:

- Qatar: Growth Factor Study (Clinical Trials.gov NCT 01812564)
- Qatar: Hamstring Acute Rehabilitation Study (Clinical Trials.gov NCT02104258)
- The Netherlands: Hamstring Injection Therapy Study (Dutch Trial Register 2771)
- The Netherlands: Diffuse Tensor Imaging (DTI) for Hamstring Injury Study (Dutch Trial Register 2016_033)

Data Cleaning Protocol

I. All new variables were derived/recoded from merged dataset (e.g., variables differentiating for injured and uninjured leg derived from the injured side and variables for right and left leg).

II. Manually correcting all odd data points as found during the verifying process.

III. Manually check the listed recoding formulas for coding errors.

IV. Manually checking all measurements of a selection of 3% of all participants per database in a statistical software package to check for consistency with original measurements after performing pre-listed recoding formulas. When the percentage of disagreement exceeds 1.5% within a measurement of one-time point, the random selection is increased to 15% of all participants for that measurement. If the percentage of disagreement exceeds 1.5% within the measurements of one-time point in the extended selection of participants, all measurements from that time point will be digitally rescanned and data will be reprocessed. All indicated faults will be corrected in the original data sets.

V. Checking data of each variable is within possible ranges and manually correcting data points based on the original study forms.

VI. Checking data on logic inconsistencies, i.e., checking dates of time points of date of injury, first visit, time of MRI, RTP, and reinjury for chronologic consistency.

Data cleaning log

- Randomly "baseline variables" data of***
  - 3 out of 90 (3%) subjects of GF Study (subject GF18, GF32, GF66)
  - 3 out of 88 (3%) subjects of HAR study) (subject HAR 20, HAR49, HAR54)
  - 2 out of 80 (3%) subjects of HIT study (subjects HIT 125 and HIT 145)
  - 4 out of 120 (3%) HIR study (subjects HIR 41, HIR 45, HIR 49, HIR 146)

are checked and compared to the source data (original database) by a colleague researcher, not involved in the process of data cleaning for the reviewed selection of data (Mokkenstorm & Zein).

Two data points were not consistent with the source data after scored twice of a total number of 4476 data points checked. This gives an error margin of 0.04%

Error margin: 2 / 4476 = 0,04%

Changes in raw data file after performing data cleaning protocol:

**Subject**	**Variable**	**False outcome**	**Source data**
GF18	All data is consistent with the original data files.		
GF32	All data is consistent with the original data files.		
GF66	All data is consistent with the original data files.		
HAR 20	All data is consistent with the original data files.		
HAR49	BaslinePainPalpationYN [Column GD]	2	1
HAR54	All data is consistent with the original data files.		
HIT 125	BaselineReceivedTreatment_Medication_Yes_No (col BX)	0	2
HIT 145	All data is consistent with the original data files.		
HIR 41	All data is consistent with the original data files.		
HIR 45	All data is consistent with the original data files.		
HIR 49	All data is consistent with the original data files.		
HIR 146	All data is consistent with the original data files.		

Supplement Appendix 4: Descriptive baseline statistics for total study population and separated into those who did or did not incur a reinjury within 2- and 12 months return to play.

Demographics & History	Total population (n= 368)	2 months reinjury (n= 330)		12 months reinjury (n= 308)	
		No reinjury (n= 299; 91%)	Reinjury (n= 31; 9%)	No reinjury (n= 256; 83%)	Reinjury (n= 52; 17%)
**Age (year)***	26 (21-30)	26 (21-30)	28 (23-30)	26 (21-30)	26 (22-30)
**Sex male; n (%)**	357 (97.0%)	290 (97.0%)	29 (93.5%)	250 (97.7%)	49 (94.2%)
**Side of hamstring injury; n (%)**					
- Right	199 (54.0%)	156 (52.2%)	19 (61.3%)	134 (52.3%)	29 (55.8%)
- Left	169 (46.0%)	143 (47.8%)	12 (38.7%)	122 (47.7%)	23 (44.2%)
**Days since injury (day)***	3 (2-4)	3 (2-4)	3 (2-5)	3 (2-4)	3 (2-4)
**Height (meter)***	1.790 (1.740-1.840)	1.790 (1.740-1.840)	1.800 (1.710-1.860)	1.790 (1.740-1.840)	1.805 (1.725-1.852)
**Weight (kg)***	75.500 (70-80)	75.700 (70.000 – 84.000)	76.000 (71.200-82.050)	75.000 (70.000-83.250)	75.500 (70.000-82.025)
**Body Mass Index (kg/m²)***	23.525 (22.300-25.200)	23.500 (22.340-25.200)	23.800 (22.600-24.700)	23.500 (22.300-25.240)	23.720 (22.575-24.700)
**Type of sports; n (%)**					
- Football	255 (61%)	208 (70%)	25 (81%)	178 (70%)	42 (80.8%)
- Field hockey	26 (7%)	22 (7%)	1 (3%)	20 (8%)	3 (6%)
- Athletics	17 (5%)	13 (4%)	1 (3%)	11 (4%)	2 (4%)
- Futsal	14 (4%)	13 (4%)	-	11 (4%)	1 (2%)
- Handball	11 (3%)	7 (2%)	1 (3%)	6 (2%)	1 (2%)
- Basketball	10 (3%)	9 (3%)	1 (3%)	6 (2%)	1 (2%)
- Other / Unknown	31 (8%)	27 (9%)	2 (7%)	24 (10%)	2 (4%)
**Level of Sports; n (%)**					
- Professional	241 (65%)	194 (65%)	19 (61%)	168 (66%)	29 (56%)

- Non-professional (Recreational/competitive)	121 (33%)	102 (34%)	11 (36%)	85 (33%)	22 (42%)
- Unknown/missing	6 (2%)	3 (1%)	1 (3%)	3 (1%)	1 (2%)
**History of hamstring injury; n (%)**					
- Yes	181 (49.2%)	146 (48.8%)	17 (54.8%)	120 (46.9%)	32 (61.5%)
- No	149 (40.5%)	125 (41.8%)	9 (29.0%)	110 (43.0%)	14 (26.9%)
- Unknown/missing	38 (10.3%)	28 (9.4%)	5 (16.1%)	26 (10.2%)	6 (11.5%)
**History of hamstring injury on ipsilateral leg; n (%)**					
- Yes	132 (35.9%)	107 (35.8%)	14 (45.2%)	88 (34.4%)	26 (50%)
- No	198 (53.8%)	164 (54.8%)	12 (38.7%)	142 (55.5%)	20 (38.5%)
- Unknown/missing	38 (10.3%)	28 (9.4%)	5 (16.1%)	26 (10.2%)	6 (11.5%)
**Moment of injury; n (%)**					
- Match/Competition	246 (66.8%)	198 (66.2%)	23 (74.2%)	169 (66%)	38 (73.1%)
- Training	112 (30.4%)	94 (31.4%)	7 (22.6%)	80 (31.3%)	13 (25%)
- Unknown/missing	10 (2.7%)	7 (2.3%)	1 (3.2%)	7 (2.7%)	1 (1.9%)
**Injury Mechanism: Sprinting / non-sprinting; n (%)**					
- Sprinting	101 (27.4%)	82 (27.4%)	10 (32.3%)	67 (26.2%)	20 (38.5%)
- Non-sprinting	265 (72.0%)	215 (71.9%)	21 (67.7%)	187 (73%)	32 (61.5%)
- Unknown/missing	2 (0.5%)	2 (0.7%)	-	2 (0.8%)	-

**Physical examination**	**Total population (n= 368)**	**2 months reinjury (n= 330)**		**12 months reinjury (n= 308)**	
		**No reinjury (n=299; 91%)**	**Reinjury (n=31; 9%)**	**No reinjury (n= 256; 83%)**	**Reinjury (n= 52; 17%)**
**Length of painful area (cm)***	8.000 (5.000-12.000)	8.000(5.000-12.000)	8.000 (3.000-12.500)	8.000 (5.000-13.000)	8.000 (4.500-11.500)
**Discomfort restricted flexion 90 degrees; n (%)**					
- Present	287 (78.0%)	230 (76.9%)	25 (80.6%)	194 (75.8%)	43 (82.7%)
- Absent	56 (15.2%)	45 (15.1%)	6 (19.4%)	39 (15.2%)	8 (15.4%)
- Unknown	25 (6.8%)	24 (8.0%)	-	23 (9%)	1 (1.9%)

**Discomfort during Active Knee Extension; n (%)**					
- Present	123 (33.4%)	95 (31.8%)	17 (54.8%)	79 (30.9%)	26 (50%)
- Absent	147 (39.9%)	118 (39.5%)	9 (29.0%)	96 (37.5%)	17 (32.7%)
- Unknown	98 (26.6%)	86 (28.8%)	5 (16.1%)	81 (31.6%)	9 (17.3%)
**Active knee extension test (degree)***					
- Injured leg angle (degrees)	65.000 (40.000-105.500)	66.000 (41.650-109.000)	82.500 (41.000-128.000)	65.000 (40.000-102.000)	82.500 (42.500-128.00)
- Uninjured leg angle (degrees)	84.600 (67.000-121.250)	85.000 (70.000-125.000)	90.000 (62.250-138.500)	85.000 (70.000-121.250)	90.000 (67.000-140.000)
- Deficit in flexibility (degrees)	15.000 (4.750-33.050)	15.000 (4.000-33.600)	11.000 (3.250-26.500)	13.000 (4.000-33.500)	18.500 (5.000-28.500)
**Passive straight leg raise test (degree)**					
- Injured leg angle (degrees)**	70.473 (±17.256)	70.390 (±17.420)	63.621 (±17.885)	69.732 (±17.127)	65.082 (±17.560)
- Uninjured leg angle (degrees)*	60.000 (50.000-72.000)	62.000 (50.000-72.000)	55.000 (50.000-61.000)	62.000 (50.000-72.000)	56.000 (50.000-68.000)
- Deficit in flexibility (degrees)*	10.000 (2.000-23.000)	13.927 (±15.778)	10.000 (2.000-20.000)	10.000 (2.000-23.500)	8.000 (0-20.000)
**Isometric knee flexion resistance force in 15° (Newton)***					
- Injured leg (Newton)**	145.254 (±79.507)	145.764 (±79.746)	138.686 (±79.660)	145.401 (±78.703)	141.202 (±82.233)
- Uninjured leg (Newton)*	243.000 (203.400-287.000)	247.100 (205.000-289.300)	227.500 (196.00-.276.300)	245.200 (202.700-287.150)	240.100 (199.000-278.600)
- force deficit (Newton)*	94.200 (42.325-150.100)	97.100 (43.000-149.550)	72.000 (40.950-183.400)	94.200 (41.100-149.100)	98.550 (44.425-179.475)
**Isometric knee flexion resistance force in 90° (Newton)**					
- Injured leg (Newton)**	134.761 (±54.628)	134.683(±54.986)	147.314 (±54.330)	133.036 (±54.337)	147.306 (±49.814)
- Uninjured leg (Newton)**	176.611 (±48.563)	176.766 (±48.883)	186.224 (±55.326)	174.134 (±48.471)	187.355 (±49.323)
- force deficit (Newton)*	37.300 (5.000-74.575)	37.300 (3.900-75.000)	20.800 (5.600-81.550)	37.300 (2.00-76.500)	23.500 (8.700-67.175)
**Return to play**	**Total population (n= 368)**	**2 months reinjury (n= 330)**		**12 months reinjury (n= 308)**	
		**No reinjury (n=299; 91%)**	**Reinjury (n=31; 9%)**	**No reinjury (n=256; 83%)**	**Reinjury (n=52; 17%)**
**Time to RTP (days)***	31 (21-46)	31 (21-46)	32.50 (19.25-44.50)	31 (21-48.50)	31 (19-42)
**MRI findings**	**Total population (n= 368)**	**2 months reinjury (n= 330)**		**12 months reinjury (n= 308)**	
		**No reinjury**	**Reinjury**	**No reinjury**	**Reinjury**

		(n=299; 91%)	(n=31; 9%)	(n=256; 83%)	(n=52; 17%)
**Involved muscles; n (%)**					
- Biceps Femoris	284 (77.2%)	231 (77.3%)	24 (77.4%)	194 (75.8%)	44 (84.6%)
- Semimembranosus/Semitendinosus	84 (22.8%)	68 (22.7%)	7 (22.6%)	62 (24.2%)	8 (15.4%)
**Modified Peetrons grading; n (%)**					
- Grade 1	126 (34.2%)	100 (33.4%)	10 (32.3%)	85 (33.2%)	17 (32.7%)
- Grade 2	242 (65.8%)	199 (66.6%)	21 (67.7%)	171 (66.8%)	35 (67.3%)
**Tendon involvement; n (%)**					
- No tendon involvement	173 (47.0%)	140 (46.8%)	14 (45.2%)	118 (46.1%)	26 (50%)
- Tendon involvement	195 (53.0%)	159 (53.2%)	17 (54.8%)	138 (53.9%)	26 (50%)
**MTJ involvement; n (%)**					
- No MTJ involvement	104 (28.3%)	90 (30.1%)	4 (12.9%)	79 (30.9%)	8 (15.4%)
- MTJ involvement	264 (71.7%)	209 (69.9%)	27 (87.1%)	177 (69.1%)	44 (84.6%)
**Extent of oedema (cm)***					
- Anteroposterior (cm)	1.900 (1.100-3.095)	1.800 (1.100-3.020)	2.000 (1.190-3.020)	1.800 (1.100-3.200)	1.500 (0.935-2.590)
- Transverse (cm)	2.200 (1.400-3.200)	2.200 (1.440-3.125)	1.855 (1.050-3.275)	2.230 (1.400-3.200)	2.020 (1.410-3.200)
- Craniocaudal (cm)	13.200 (8.300-20.000)	13.000 (8.238-19.925)	13.750 (7.275-16.800)	13.200 (8.200-19.900)	13.000 (7.700-16.225)
**Extent of haematoma (cm)***					
- Anteroposterior	0.000 (0.000-0.700)	0.000 (0.00-0.700)	0.550 (0.000-0.900)	0.000 (0.000-0.600)	0.500 (0.000-0.800)
- Transverse	0.000 (0.000-0.820)	0.000 (0.00-0.810)	0.620 (0.000-0.955)	0.000 (0.000-0.810)	0.600 (0.000-0.900)
- Craniocaudal	0.000 (0.000-2.475)	0.000 (0.000-2.400)	1.700 (0.000-3.000)	0.000 (0.000-2.300)	1.500 (0.000-3.000)
**IM tendon disruption; n (%)**					
- No IM tendon disruption	183 (45.7%)	146 (48.2%)	13 (41.9%)	129 (50.4%)	23 (44.2%)
- IM tendon disruption	168 (49.7%)	146 (48.8%)	17 (54.8%)	124 (48.4%)	28 (53.8%)
- Unknown	17 (4.6%)	9 (3%)	1 (3.2%)	3 (1.2%)	1 (1.9%)
**Complete IM tendon disruption; n (%)**					
- No complete disruption	324 (88.0%)	266 (89%)	29 (93.5%)	232 (90.6%)	49 (94.2%)

- Complete disruption	27 (7.3%)	24 (8%)	1 (3.2%)	21 (8.2%)	2 (3.8%)
- Unknown	17 (4.6%)	9 (3%)	1 (3.2%)	3 (1.2%)	1 (1.9%)
**Length of IM tendon disruption (cm)***	0.000 (0.000-5.600)	0.000 (0.000-5.600)	1.500 (0.000-5.225)	3.202 (0.000-5.650)	2.727 (0.000-4.800)
**Presence of waviness; n (%)**					
- No present	216 (58.7%)	178 (59.5%)	18 (58.1%)	152 (59.4%)	37 (71.2%)
- Present	131 (35.6%)	109 (36.5%)	11 (35.5%)	98 (38.3%)	13 (25%)
- Unknown	21 (5.7%)	12 (4%)	2 (6.5%)	6 (2.3%)	2 (3.8%)
**Presence of fibrosis; n (%)**					
- No present	322 (87.5%)	269 (90%)	26 (83.9%)	231 (90.2%)	45 (86.5%)
- Present	32 (8.7%)	23 (7.7%)	4 (12.9%)	20 (7.8%)	6 (11.5%)
- Unknown	14 (3.8%)	7 (2.3%)	1 (3.3%)	5 (2%)	1 (1.9%)

Abbreviations: IQR = Interquartile range; SD = Standard Deviation; RTP = return to play, MTJ = myotendinous junction, IM = Intramuscular.
*Presented in median (IQR).
**Presented in mean (SD).

References

1. Hamilton, B. *et al.* Platelet-rich plasma does not enhance return to play in hamstring injuries: a randomised controlled trial. *Br. J. Sports Med.* **49**, 943–950 (2015).

2. Vermeulen, R. *et al.* Early versus delayed lengthening exercises for acute hamstring injury in male athletes: a randomised controlled clinical trial. *Br. J. Sports Med.* **56**, 792 LP – 800 (2022).

3. Reurink, G. *et al.* Platelet-rich plasma injections in acute muscle injury. *The New England journal of medicine* **370**, 2546–2547 (2014).

4. Heiderscheit, B. C., Sherry, M. A., Silder, A., Chumanov, E. S. & Thelen, D. G. Hamstring strain injuries: recommendations for diagnosis, rehabilitation, and injury prevention. *J. Orthop. Sports Phys. Ther.* **40**, 67–81 (2010).

5. Sherry, M. A. & Best, T. M. A comparison of 2 rehabilitation programs in the treatment of acute hamstring strains. *J. Orthop. Sports Phys. Ther.* **34**, 116–125 (2004).

6. Askling, C. M., Tengvar, M., Tarassova, O. & Thorstensson, A. Acute hamstring injuries in Swedish elite sprinters and jumpers: a prospective randomised controlled clinical trial comparing two rehabilitation protocols. *Br. J. Sports Med.* **48**, 532–539 (2014).

7. Askling, C. M., Tengvar, M. & Thorstensson, A. Acute hamstring injuries in Swedish elite football: a prospective randomised controlled clinical trial comparing two rehabilitation protocols. *Br. J. Sports Med.* **47**, 953–959 (2013).

8. Whiteley, R., van Dyk, N., Wangensteen, A. & Hansen, C. Clinical implications from daily physiotherapy examination of 131 acute hamstring injuries and their association with running speed and rehabilitation progression. *Br. J. Sports Med.* **52**, 303–310 (2018).

9. Tol, J. L. *et al.* At return to play following hamstring injury the majority of professional football players have residual isokinetic deficits. *Br. J. Sports Med.* **48**, 1364–1369 (2014).

10. Mendiguchia, J. *et al.* A Multifactorial, Criteria-based Progressive Algorithm for Hamstring Injury Treatment. *Med. Sci. Sports Exerc.* **49**, 1482–1492 (2017).
